# Design and Validation of an Augmented Reality Teaching System for Primary Logic Programming Education

**DOI:** 10.3390/s22010389

**Published:** 2022-01-05

**Authors:** Chi-Yi Tsai, Yu-Cheng Lai

**Affiliations:** Department of Electrical and Computer Engineering, TamKang University, 151 Yingzhuan Road, Tamsui District, New Taipei City 251, Taiwan; 403500076@gms.tku.edu.tw

**Keywords:** augmented reality, logic programming teaching, learning effectiveness, learning motivation, analysis of covariance

## Abstract

Programming is a skill that requires high levels of logical thinking and problem-solving abilities. According to the Curriculum Guidelines for the 12-Year Basic Education currently implemented in Taiwan, programming has been included in the mandatory courses of middle and high schools. Nevertheless, the guidelines simply recommend that elementary schools conduct fundamental instructions in related fields during alternative learning periods. This may result in the problem of a rough transition in programming learning for middle school freshmen. To alleviate this problem, this study proposes an augmented reality (AR) logic programming teaching system that combines AR technologies and game-based teaching material designs on the basis of the fundamental concepts for seventh-grade structured programming. This system can serve as an articulation curriculum for logic programming in primary education. Thus, students are able to develop basic programming logic concepts through AR technologies by performing simple command programming. This study conducted an experiment using the factor-based quasi-experimental research design and questionnaire survey method, with 42 fifth and sixth graders enrolled as the experimental subjects. The statistical analysis showed the following results: In terms of learning effectiveness, both AR-based and traditional learning groups displayed a significant performance. However, of the two groups, the former achieved more significant effectiveness in the posttest results. Regarding learning motivation, according to the evaluation results of the Attention, Relevance, Confidence, and Satisfaction (ARCS) motivation model, the AR-based learning group manifested significantly higher levels of learning motivation than the traditional learning group, with particularly significant differences observed in the dimension of Attention. Therefore, the experimental results validate that the proposed AR-based logic programming teaching system has significant positive effects on enhancing students’ learning effectiveness and motivation.

## 1. Introduction

Digital information technology has been perceived as an essential component of human society. In a highly digital environment, information science education has been gaining increasing importance. Accordingly, governments worldwide have begun promoting all kinds of information science education. Computational thinking has been identified as a significant core of information science education [1]. It enables learners to adopt the mindset used by computer scientists in solving problems [2]; it is also a critical core competency in programming. In recent years, increasingly more countries have included computational thinking in compulsory education [3], and the most effective way to build computational thinking is to study computer science [4]. Learning how to write programs can effectively improve students’ logical thinking, abstract reasoning, and problem-solving abilities [5]. In addition, some studies have suggested that the appropriate integration of programming concepts into primary and secondary education may help improve students’ learning motivation and efficiency in programming learning. Therefore, programming should be a skill that everyone needs to possess. Through systematic teaching, programming can help students improve their logical thinking, abstract reasoning, and problem-solving abilities, and even improve their concentration. After completing the studies, students may apply these computational thinking skills to solve problems in other fields or practical problems in real life. In order to enhance the national competitiveness in information technology, countries worldwide have included programming education as a mandatory course. Many western countries such as the United Kingdom even incorporated programming education into the compulsory curriculum plan for primary and secondary schools very early. This shows that programming and computational thinking are perceived internationally as key skills that need to be developed.

In recent years, Taiwan has also been devoting increasing attention to programming education. As such, the 2019 Curriculum Guidelines included ‘programming’ in science and technology, and specified formal learning hours for the junior and senior high school stages. Because the knowledge involved in programming languages is relatively abstract and abstruse, beginners are more likely to encounter difficulties in learning, hence reducing learning motivation [6]. Thus, many programming learning tools and software sets provide a more user-friendly environment through the use of visual programming languages to increase the interest of beginners. According to the literature [2], programming teaching that avoids using complex syntactic structures and provides connections between specific scenarios and abstract concepts is more likely to improve learners’ understanding and interest in programming. However, in the implementation of programming courses, many practical problems still need to be resolved. For example, Shi proposed the following possible challenges in programming education [7]:

1. Teacher resources: This is the primary challenge faced by most counties and cities. Even in counties and cities that advocate programming courses in primary and secondary schools, teacher resources are still a challenge;
2. Suitability of teaching materials: With rapid development of science and technology, if teachers simply lecture using paper teaching materials, there may be a disconnect from the current development of industrial technology;
3. Insufficient information equipment: Programming relies on a large number of information equipment. Schools in rural and remote areas have relatively insufficient resources in this regard, and information equipment is relatively backward;
4. Articulation of curricula: If students start to learn programming without any foundation, the learning process may add some burden on their study load. In addition to the regular basic courses, they also must learn and practice programming.

As a result, for the implementation of programming education in Taiwan as per the 2019 Curriculum Guidelines, in the future, problems regarding teacher training, teaching material design, equipment prevalence and student curriculum articulation are to be resolved. Based on these possible challenges, this paper addresses the following research questions (RQs).

**RQ1:** Does the augmented reality (AR) based teaching system effectively help teachers improve students’ learning effectiveness in primary logic programming courses?

In response to the era of artificial intelligence (AI), Taiwan’s Ministry of Education (MOE) launched the General Implementation Strategy for Artificial Intelligence and New Technology Education in 2019. The MOE published the AI education plan for students at all levels from primary school to university and announced the adjustment to include ‘machine learning’ and ‘artificial intelligence’ as required courses in the pre-service teacher training program [8]. The MOE also launched pilot projects at 386 schools of all levels across the island through pilot schools in the area of science and technology, and schools for the promotion of emerging technologies among others. However, even with these pilot schools, a wide gap in the resource of teachers and learning environments between rural and urban areas remains [9]. As such, the problem of insufficient teachers for logic programming courses continues to exist in Taiwan. In recent years, some AR-based teaching systems have been applied to natural science courses in elementary schools to improve students’ learning effectiveness [10,11]. Therefore, this article examines whether the AR-based teaching system can help teachers improve students’ learning effectiveness in primary logic programming courses.

**RQ2:** Does the AR-based teaching materials effectively stimulate students’ learning motivation in primary logic programming courses?

In the design of teaching materials, the literature [12] noted that if the teaching materials developed using any kind of instructional design cannot stimulate learners’ interest or concentration; the learners’ learning effectiveness will be greatly reduced. Therefore, ‘how to arouse learning motivation’ is a topic of profound importance in the design of teaching content. In recent years, ‘play’ has become a critical keyword in innovative education. Various studies have shown that integrating games into courses is a significant factor affecting children’s physical and mental development, facilitating their socialization, and stimulating creativity and learning motivation [13,14]. In game-based education, teachers use game mechanisms or elements to supplement their teaching content or aids. By doing so, they guide learners to actively engage in a given learning activity and keep them interested in and focused on it during their gaming experience, thereby enabling them to achieve better learning effectiveness. Learning motivation is the inner drive that triggers and sustains learning activities through the learning process. It is a psychological process in which learners are prompted to spontaneously devote their time and effort toward predetermined learning objectives during learning activities [15]. On this basis, the learning effectiveness of learners is affected to a considerable extent by the level of learning motivation in the learning process. Therefore, this article also examines whether the AR-based gamified teaching materials can effectively stimulate students’ learning motivation in primary logic programming courses.

**RQ3:** Does the AR-based teaching system simplify teaching equipment used in primary logic programming courses?

Regarding teaching equipment, with the development of technology, smartphones and wireless networks have become increasingly prevalent. They have gradually become indispensable tools in modern life. In addition, with the improvement in mobile phone development technologies and the enhancement of hardware and software equipment, many AR or virtual reality (VR) mobile applications have emerged. Nowadays, almost everyone has a smartphone. With such a high prevalence, the entry barriers for AR and VR have been greatly lowered. AR and VR technologies enable users to operate and experience virtual objects through devices, giving them access to businesses and experiences that are normally not readily accessible. Many instances of applying AR or VR technologies to education have been observed domestically and abroad [10,11,14,16]. Because AR and VR-based teaching methods may easily increase users’ learning interest and concentration, with the increasing popularity of smartphones and wireless networks, learners will have more opportunities to experience AR-based teaching without computer equipment.

Based on the aforementioned challenges for programming education development and the relevant literature review, this study proposes a novel AR-based teaching system for basic logic programming, which combines AR technology and gamified designs of teaching materials on the basis of the fundamental concepts of the seventh-grade structured programming articulation curriculum [17]. The combination of AR technologies and gamified teaching materials to develop the proposed system helps to improve learners’ interest and concentration during the learning process through AR and gamified teaching content, thereby achieving better learning results. Simply by playing AR-based mobile games, learners can achieve learning tasks and experience the thinking mode involved in logic programming. Finally, after learners experience AR-based learning and traditional learning using textbooks, the study explores their respective impacts on the learning effectiveness and motivation of logic programming based on the experimental results. In response to the above research questions, this study provides the following three major contributions:

1. In terms of assisting teachers in teaching, this study proposes an AR-based teaching system that allows users to learn logic programming through smartphones, thereby transforming teachers who originally served as the chief channel of knowledge impartation into a learning facilitator;
2. In terms of teaching material design, this study validates that adding AR functions and appropriate gamification elements to the logic programming courses can effectively stimulate students’ learning motivation;
3. In terms of simplifying teaching equipment, the proposed AR-based logic programming teaching system frees the learning of logic programming from the limitations of computer equipment. Students can learn with smartphones, which are very popular and easy to use.

The experimental results are as follows. In terms of learning effectiveness, both AR-based and traditional learning groups showed significant performance in the learning of logic programming. Of the two groups, the AR-based learning group achieved more significant learning effectiveness. Regarding learning motivation, according to the evaluation results of the ARCS Learning Motivation Questionnaire, the AR-based learning group manifested significantly higher levels of learning motivation than the traditional learning group, with particularly significant differences observed in the dimension of Attention.

## 2. Research Method

This study adopted a factorial experimental design to examine the differences in the learning motivation and learning effectiveness of eleven and twelve-year-old students who learned logic programming using the traditional paper-based learning method and those who used the AR-based learning method. The experimental subjects, research design, learning activities, and research instruments are explained below.

### 2.1. Participants

The subjects of this experiment are mainly eleven and twelve-year-old students in two classes of a primary school in Hsinchu County. Students in each class were randomly grouped into AR-based and traditional learning groups for logic programming training. The total numbers of participants were 22 and 20 for the AR-based and traditional learning groups, respectively. The two classes had equal numbers of participants for both groups, i.e., 11 and 10 each for the AR-based and traditional learning groups, respectively. Note that, since there is no logic programming course in the formal curriculum of elementary schools, most of the participants have no relevant logic programming experience. In the pretest questionnaire, of the 42 students who participated in the experiment, 34 had never studied logic programming before.

### 2.2. Design

This study employed the self-developed mobile application, the AR-based logic programming teaching system, as a teaching aid for basic programming logic learning and cultivation. In terms of learning effectiveness, the analysis of covariance (ANCOVA) was employed as the experimental design method. The independent variables were the experimental subjects in the AR-based and traditional learning groups, whereas the pretest and posttest surveys were, respectively, used as the covariate and dependent variables of the analysis. Regarding learning motivation, the analysis of variance (ANOVA) was adopted as the experimental design method. The independent variables were the experimental subjects in the AR-based and traditional learning groups; the control variables comprised teaching methods, teaching materials, instructors, learning time, and pretest and posttest questions and the dependent variables were the four fundamental dimensions of the ARCS motivation model and theory proposed by M.J. Keller [18]: Attention, Relevance, Confidence and Satisfaction. In this study, the experimental data were collected through quasi-experimental research and questionnaire survey methods. After the experiment, a quantitative analysis was conducted based on the survey results regarding the learning effectiveness and motivation of the two groups.

#### 2.2.1. Questionnaire Survey

This study employed the Learning Effectiveness Questionnaire (LEQ) and the ARCS Learning Motivation Questionnaire (LMQ) as experiment questionnaires. Two groups of subjects were tested with two different logic program teaching aids and the same logic program teaching method. The two groups were required to receive pretests and posttests and fill out questionnaires.

#### 2.2.2. Questionnaire Analysis Method

This study conducted a data analysis using IBM SPSS Statistics 28.0.0 to find out the effect of the AR-based teaching system for basic logic programming on students’ learning effectiveness and learning motivation. The results of the LEQ were analyzed using the ANCOVA approach. With the initial differences in students’ backgrounds removed, the effects of the teaching models on students’ learning effectiveness were examined. The data of the ARCS LMQ were analyzed using the ANOVA approach to compare the mean differences in the learning motivation of the two groups of students.

### 2.3. Teaching Experiment

The total time of the teaching experiment in this study was 50 min. The experiment process included an activity description and random grouping (approximately 5 min), pretest administration of the LEQ (approximately 10 min), logic programming curriculum experience (approximately 20 min), and posttest administration of the LEQ and administration of the ARCS LMQ (approximately 15 min).

### 2.4. Research Instruments

A total of three questionnaires were used in this study, i.e., the LEQ pretest, the LEQ posttest and the ARCS LMQ. Among these three, the LEQ pretest was administered before the logic programming training activity, and the other two were administered after the activity.

#### 2.4.1. Learning Effectiveness Questionnaire

As a method to assess learning effectiveness in this study, the LEQ was divided into four sections:

1. The filling of demographic information;
2. The logic programming integrating into life issues;
3. The logic programming concepts;
4. The application of logic programming concepts.

This study preserves anonymity to protect the personal information of the subjects. Therefore, the experiment subjects only need to provide their grade, group code and number for the demographic information in the first section. All data were provided as statistical data and for academic research use only. The second to fourth sections are test papers. Through the test of logic programming questions, the study examined whether the subjects had improved concepts of logic programming after the activity. The test questions were designed for the following three dimensions, seeking to understand the level of subjects’ comprehension and application of logic programming.

1. Logic programming integrated with life issues;
2. Logic programming concepts;
3. Application of logic programming concepts;

#### 2.4.2. ARCS Learning Motivation Questionnaire

The ARCS LMQ used in this study was based mainly on the ARCS motivation model proposed by John Keller and was specifically designed for the content of this thesis with references taken from the following ARCS-related academic theses [19,20,21]. The questionnaire is divided into four dimensions in accordance with the theory, namely, Attention, Relevance, Confidence, and Satisfaction. Each dimension comprises five questions with negatively worded items included in each dimension, as shown in Table 1. On the basis of the responses to the negatively worded items, the questionnaires randomly answered by the subjects were classified as invalid samples. Designed in accordance with the Likert scale, the questionnaire contained the options of Strongly Agree, Agree, Neutral, Disagree, and Strongly Disagree. The positively worded items were scored in the order of 5, 4, 3, 2, and 1, whereas the negatively worded items were scored in the order of 1, 2, 3, 4, and 5. The higher the score was, the higher the degree of agreement the respondent showed.

According to the reliability analysis of the ARCS LMQ data collected from the experiment, the ARCS LMQ achieved an overall reliability of 0.932. The Cronbach’s α value for the four dimensions, Attention, Relevance, Confidence and Satisfaction, arrived at 0.861, 0.889, 0.850, and 0.851 respectively, which indicated a medium-to-high level of reliability.

## 3. The Proposed System

This section explains the teaching concept of the proposed AR-based logic programming teaching system and introduces the functions of this teaching system and the focus of each learning topic.

### 3.1. Concept of the Proposed AR-Based Logic Programming Teaching System

**Figure 1:**
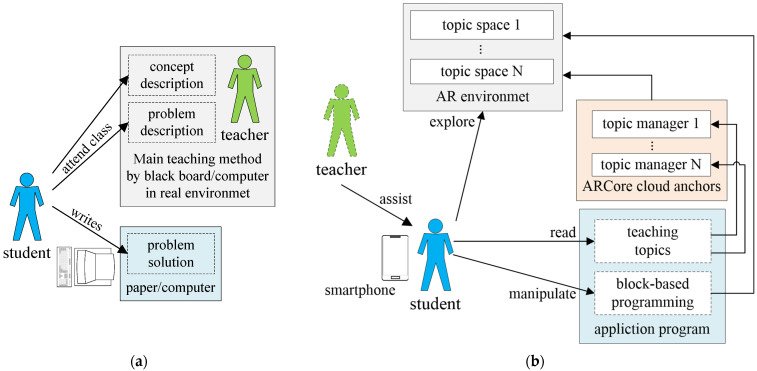
Comparison between (**a**) the traditional logic programming teaching method and (**b**) the proposed AR-based logic programming teaching method. In traditional logic programming teaching, teachers mainly teach concepts and describe problems through lectures, while students solve and verify problems through program coding. In the proposed AR-based logic programming teaching, students play a crucial and active role in operating the teaching system, and teachers serve the role of learning facilitators in teaching.

The AR-based teaching system for basic logic programming proposed in this study offers students a different learning method from traditional logic programming teaching methods. The differences between the two are shown in Figure 1. Figure 1a displays the traditional logic programming teaching, and Figure 1b presents the AR-based logic programming teaching proposed in this study. In traditional logic programming teaching, teachers mainly teach concepts and describe problems through lectures, while students solve and verify problems through program coding. In the proposed AR-based logic programming teaching, students play a crucial and active role in operating the teaching system, whereas teachers serve the role of learning facilitators in teaching. Simply by selecting a learning topic through the mobile application, students can immediately read the fundamental knowledge of the selected topic and enter the AR-based environment for the topic to learn related knowledge. After receiving a problem description related to the learning topic in the AR environment, students execute logic command block programming on their smartphones to control the virtual objects in the AR environment and directly perform functional testing and verification in the AR environment. Therefore, the proposed AR-based teaching system can concretize the logic programming training process.

### 3.2. Platform

**Figure 2:**
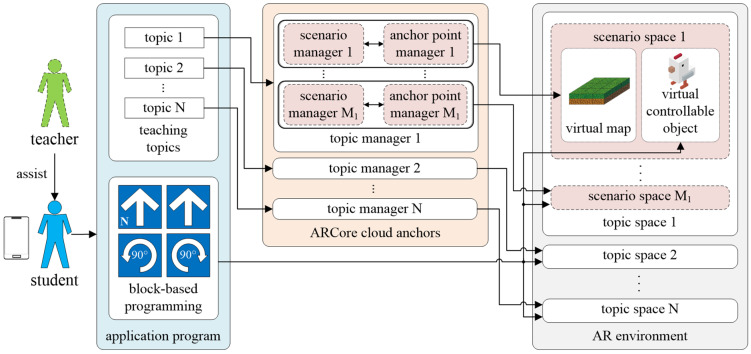
Architecture of the proposed teaching system, which comprises three parts: the application program, ARCore cloud anchors, and AR environment. The application program contains multiple teaching topics, each of which is implemented by the AR-based teaching content in the system. The ARCore cloud anchor module contains the same number of topic managers as topics. Each topic manager has multiple scene managers and anchor managers to store virtual contents and the corresponding anchor positions, respectively. The AR environment is composed of multiple topic spaces, each of which comprises multiple scenario spaces related to learning topics.

The proposed logic programming teaching system is a mobile application developed for the Android system. It enables students to autonomously learn the concepts of logic programming and operate in a visualized manner in the AR environment. Figure 2 displays the architecture of the proposed teaching system, which comprises three parts: the application program, ARCore cloud anchors, and AR environment. The application program features two key functions. One is the teaching topic menu, which is mainly used to connect the AR-based teaching content in the system. When a topic is selected, the system will load the scene to the anchor position temporarily pre-stored in the topic space according to the selected topic via the topic manager in the ARCore cloud anchor. The other is the function of block-based programming, which is mainly used to control the virtual controllable objects in the scene space for the implementation of basic logic programming learning. The ARCore cloud anchor module contains the same number of topic managers as topics. Each topic manager has multiple scene managers and anchor managers. Scene managers are primarily used to store virtual maps and objects, and anchor managers temporarily store the corresponding anchor positions of the virtual maps and objects in the real environment. The AR environment is composed of multiple topic spaces. Each topic space comprises multiple scenario spaces, each of which contains a virtual map and controllable object. The size, motion path, and destination target of each virtual map are planned in advance according to learning topics. In this regard, the results of controlling virtual objects through block programming are also displayed in the AR environment. Take the basic logic programming teaching in this study as an example. Three teaching topics are included in the system, i.e., sequence structure, selection structure, and repetition structure. Through the functions of the ARCore cloud anchor, the spaces of the three topics are arranged in different positions of the real environments for students to learn. In addition, the corresponding virtual maps are planned according to the three topics, and students may further engage in practices for corresponding logic concepts through the function of block programming.

#### 3.2.1. AR Environment

**Figure 3:**
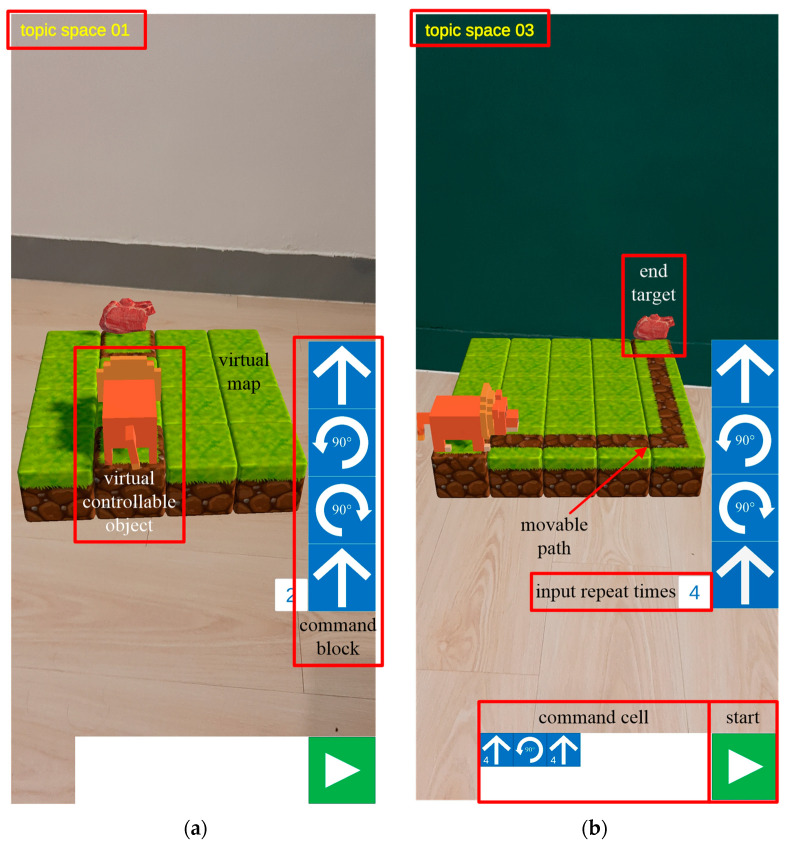
(**a**) Phone screen after opening topic 1; (**b**) phone screen after programming topic 3.

**Figure 4:**
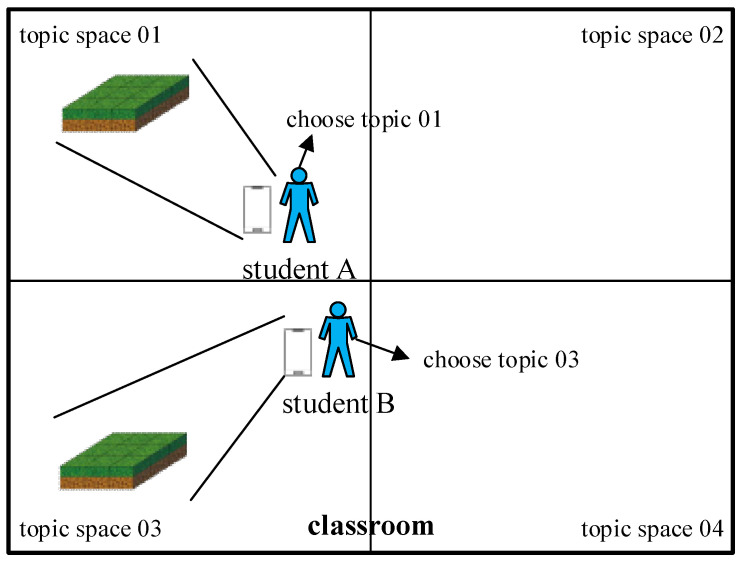
Space concept map taking the classroom as an example.

Figure 3a shows the mobile phone screenshot after Topic 1 is opened. Students can see the virtual map and object in the AR environment simply by opening Topic 1 on their smartphones and then moving to the designated location. Figure 3b displays the mobile phone screenshot after Topic 3 is opened and block programming is completed. The disparity in the backgrounds between Figure 3a,b demonstrates that different topic spaces can be set in different areas of the actual space through the ARCore cloud anchor for the implementation of autonomous learning. Students should take notice of the possible motion path the system has for the object before practicing programming and then click the command block on the right side of the screen to perform programming. The command cell at the bottom of the phone screen displays the results of the current programming. Upon completing programming, students can click the Start button on the bottom right corner of the screen, and the system will execute the actions specified in the command cell in the designated order. The system will control and start moving the virtual object in the map and determine whether the learning content in the scene space is completed through the collision between the virtual object and destination target. If students wish to modify the content of programming before clicking on Start, they can directly hit the Cancel command in the command cell. If the object cannot be correctly moved to the destination after students click on Start, the system will automatically initialize the original map and prompt them to reprogram. Figure 4 indicates a conceptual diagram of the space using the classroom as an example.

#### 3.2.2. Command Block Design

Table 2 displays the menu of command blocks supported by the proposed teaching system. The command blocks mainly control the virtual controllable objects. Students need to contemplate how to use command blocks to program actions and move objects to the correct destination positions. Table 3 shows the curriculum structure of the proposed logic programming teaching system. The curriculum comprises three learning topics: sequence, selection, and repetition structures. The three topics share common task goals, but their respective virtual map scenes, controllable virtual objects, available command blocks, and learning focuses differ.

Note that interested readers can refer to the following online video to watch the demonstration of the proposed teaching system: Demo video—https://youtu.be/-IpyBerj1C0 (accessed on 14 November 2021)

## 4. Results and Discussion

This section presents the statistical analysis conducted based on the questionnaires recovered in the experiment and discusses the analysis results of learning effectiveness and learning motivation.

### 4.1. Analysis of Learning Effectiveness

The ANCOVA framework was conducted based on the results of LEQ pretests and posttest taken by the two groups of students to whom different teaching methods were applied. A total of 42 valid samples were recovered. ANCOVA was employed to compare the differences in learning effectiveness between the two groups.

#### 4.1.1. Test of Intra-Group Homogeneity of Regression Slopes

Before ANCOVA is performed, a test should be conducted on the two groups to see whether their pretest and posttest scores show the same regression slopes. If the results of data testing fulfil the premise of ANCOVA, i.e., high intra-group homogeneity of regression coefficients, the subsequent ANCOVA can be proceeded with. The results of the test of intro-group homogeneity of regression slopes in this study are shown in Table 4.

The analysis results showed that the test of intro-group homogeneity of the learning effectiveness tests using different teaching methods did not reach the significant level of 0.05. The *F*-value achieved was 2.803, and the *p*-value of 0.102 (>0.05), hence fulfilling the premise of the ANCOVA (i.e., high intra-group regression slopes). On this basis, the study could proceed with the ANCOVA analysis.

#### 4.1.2. ANCOVA Results

Table 5 shows the descriptive statistics of the pretest and posttest results of learning effectiveness under different teaching methods. The mean score and standard deviation of the pretest in the AR-based learning group were 68.27 and 2.944, respectively, while the traditional learning group reached 71.05 and 2.924, respectively. The mean score and standard deviation of the AR-based learning group in the posttest were 91.68 and 1.545, respectively, while the traditional learning group reached 85.75 and 2.487, respectively. Note that, since most of the participants in the experiment have never learned logic programming before, the difference between the pretest and posttest mean scores is relatively large.

Table 6 presents the ANCOVA analysis results, which show that after excluding the influence of the pretest scores, the *F*-value reached 12.149, and the *p*-value reached 0.001 (<0.05). The AR-based learning group obtained higher posttest scores than the traditional learning group, indicating that when different teaching methods were employed, the students displayed significant differences in their pretest and posttest performances in terms of learning effectiveness.

According to Table 7, after excluding the influence of the pretest scores, the adjusted mean score of the AR-based learning group was 92.321 and that of the traditional teaching group was 85.047. The AR-based learning group achieved higher adjusted posttest scores than the traditional learning group, indicating that learners in the AR-based learning group performed better than those in the traditional learning group in terms of learning effectiveness.

### 4.2. Analysis of Learning Motivation

The ANOVA was conducted based on the results of the ARCS LMQ administered by the two groups of students to whom different teaching methods were applied. A total of 42 valid samples were recovered. ANOVA was adopted to compare the difference in learning motivation between the two groups. The analysis results are shown in Table 8.

#### 4.2.1. Attention

The analysis of each question in the Attention dimension is shown in Table 9. According to the analysis results, the AR-based learning group and the traditional learning group displayed extremely significant differences in all five questions. The mean scores were higher in the AR group than in the traditional group, which indicates that AR-based teaching is more effective in obtaining students’ attention than traditional teaching. The mean deviations in the five questions between the two groups fell between 1.14 and 1.34, implying that AR teaching achieved better effects than traditional teaching in attracting students’ attention.

#### 4.2.2. Relevance

The analysis of each question in the Relevance dimension is shown in Table 10. According to the analysis results, the two groups displayed extremely significant differences in Question 8, and significant differences in the remaining four questions, i.e., Questions 6, 7, 9, and 10. The AR-based learning group obtained higher mean scores than the traditional learning group in all five questions. An inference can be derived from the analysis result of Question 8 that the teaching method adopted for the AR-based learning group can enhance students’ impression of the knowledge on each topic during their logic programming topic learning experience. The mean deviations of the other four questions (Questions 6, 7, 9, and 10) fell approximately between 0.6 and 0.7, indicating that students receiving AR-based teaching generally showed higher levels of relevance to logic programming than those receiving traditional teaching.

#### 4.2.3. Confidence

The analysis of each question in the Confidence dimension is shown in Table 11. According to the analysis results, the two groups displayed significant differences in Question 14 and extremely significant differences in Questions 11, 12, 13, and 15. The AR-based learning group obtained higher mean scores than the traditional learning group in all five questions, indicating that students receiving AR-based logic programming training manifested higher levels of confidence than those receiving traditional training. The mean values of the five questions in the Confidence dimension answered by the two groups reached 4.52, 4.19, 4.29, 4.31, and 4.50. This demonstrated that the 42 students in the two groups had generally developed sufficient confidence in this new learning field after receiving logic programming training.

#### 4.2.4. Satisfaction

The analysis of each question in the Satisfaction dimension is shown in Table 12. According to the analysis results, the two groups displayed extremely significant differences in Questions 16 (a negatively worded item) and 20 and manifested significant differences in the other three questions, i.e., Questions 17, 18, and 19. The AR-based learning group obtained higher mean scores than the traditional learning group in all five questions. The majority of the students in the AR-based learning group expressed levels of satisfaction with the methods of logic programming learning and application after their learning experience higher than students in the traditional learning group. The results of the five items in the Satisfaction dimension reveal that the level of curiosity students had for this learning method was proportional to the degree of their satisfaction.

## 5. Discussion

This study developed a primary logic programming teaching system that combines AR technologies and game-based teaching materials on the basis of the fundamental concepts for seventh-grade structured programming. The proposed teaching system can serve as an articulation curriculum for logic programming in primary education. After implementation and evaluation, the statistical analysis showed that the AR-based teaching system not only achieved more significant learning effectiveness, but also enabled students to show a higher level of learning motivation than the traditional teaching method based on the evaluation results of the ARCS motivation model. Therefore, the proposed AR-based teaching system has significant positive effects on enhancing students’ learning effectiveness and motivation. Note that, the small sample size may lead to biased results in the statistical analysis. Therefore, the sample size can be identified as a limitation of this study.

Some previous studies also involved the development of teaching aids for different levels of logic programming education. For example, Evripidou et al. [14] proposed an interactive learning tool that uses educational robots to introduce algorithmic thinking and sequencing suitable for elementary and intermediate students. Vosinakis et al. [16] proposed a VR-based platform to assist in teaching Prolog programming courses in universities. The teaching platform can intuitively interpret and verify program results through VR technology and requires college students to adopt a collaborative problem-solving approach to solve problems. In contrast, the AR-based teaching system proposed in this study aims to assist the teaching of basic logic programming suitable for elementary students. Moreover, in the design of teaching materials, we combined AR technology and gamification design based on three basic programming structures, namely sequence structure, selection structure, and repetition structure. This kind of AR teaching material design not only helps to increase learners’ interest and concentration in the learning process, but also achieves better learning results in basic programming structures.

The course lecturers who participated in this study also pointed out that when students experience AR-based teaching, they are much more active in the classroom than usual; while students using traditional teaching methods are not much different from ordinary classes. Since the proposed AR-based teaching system can be easily executed on mobile devices, students can engage effective logic programming learning in both formal and informal environments. This is one of the main contributions of the proposed teaching system for students to free the learning of logic programming from the limitations of computer equipment.

## 6. Conclusions and Future Work

In response to curriculum articulation problems in the information technology area encountered during the 2019 Curriculum Guidelines implementation, this study developed an AR-based logic programming teaching system, which enables eleven and twelve-year-old students to access logic programming early, thereby lessening the pressure middle school freshmen may experience in the face of required programming courses. In order to effectively improve the learning effectiveness and learning motivation of students, the proposed teaching system implements three modules based on AR technology, namely the application program, the ARCore cloud anchor, and the AR environment. Aside from the user interface, the application program module also includes the development of the block programming function, which enables users to learn basic logic programming. The ARCore cloud anchor module aims to store the virtual maps of different learning topics and the information on their locations in the real environment so that users can learn different topics in different environments and locations. The AR environment module covers multiple topic spaces and scene spaces. Through the AR environment, the block programming results developed by users will be displayed in the scene space where the phone is located, thereby transforming logic programming learning from abstract programming to visualized execution results.

According to the experimental results and questionnaire analysis, the proposed AR-based teaching system has beneficial effects on students’ overall learning effectiveness and motivation in logic programming. In terms of learning effectiveness, although the AR-based learning group displayed greater improvements in test scores than the traditional learning group, all eleven and twelve-year-old students made significant progress in logic programming learning regardless of teaching methodology. Regarding learning motivation, because the AR-based teaching method is more likely to enhance students’ concentration and interest, the results of the ARCS LMQ revealed that the AR-based learning group obtained higher scores than the traditional learning group. The aforementioned results demonstrate that the AR-based logic programming teaching system proposed in this study has significant positive effects on enhancing students’ learning effectiveness and motivation.

There remains a lot of room for development in the future. First, constructing an experiment that divides a large number of participants into multiple groups can improve the accuracy of statistical analysis and validate the current findings. In addition to increasing the depth of logic programming learning, the command blocks can be further expanded in the aspect of block programming. In addition, this system was developed using the development kit, Unity AR Foundation, which can support multiple platforms. This advantage can be leveraged in the future to broaden the coverage of programming. For example, future research may attempt to include the function of AR connections, allowing teachers and learners to simultaneously learn and interact in the AR environment.

## Figures and Tables

**Table 1:** Distribution of ARCS questionnaire questions.

Dimension of Learning Motivation	Question Number	Reverse Question
Attention	1, 2, 3, 4, 5	1
Relevance	6, 7, 8, 9, 10	7
Confidence	11, 12, 13, 14, 15	13
Satisfaction	16, 17, 18, 19, 20	16

**Table 2:** Command function table.

Command	Function	Command	Function
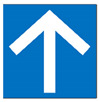	Go one block forward	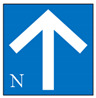	Go N block forward
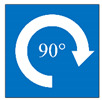	Turn right 90 degrees	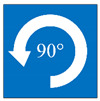	Turn left 90 degrees

**Table 3:** Curriculum Structure Table.

Theme	Mission	Learning Content		CT Concept
Topic 1: Sequence	Move the animals to the correct food through command programming	Command	Forward, Left, Right	Sequence, events, data
		Virtual generation	Mission map with basic route; An animal; The food the animal needs.	
		Learning focus	Use commands to control animals to move to the target food	
Topic 2: Selection		Command	Forward, Left, Right	Sequence, events, data, conditionals
		Virtual generation	Mission map with multiple routes; An animal; The food the animal needs and other food.	
		Learning focus	Determine the food corresponding to the animal, and use commands to control the animal to move to the target food	
Topic 3: Repetition		Command	Repeat forward, Left, Right	Sequence, events, data, conditionals, loops
		Virtual generation	Mission map with multiple routes; An animal; The food the animal needs and other food.	
		Learning focus	Simplify the original order schedule with the repeat function	

**Table 4:** Test of intra-group homogeneity of the within-group regression coefficients.

Source of Variance	Sum of Squares	Degree of Freedom	Mean Sum of Squares	*F*-Test	*p*-Value
Pretest	1769.564	1	1769.564	41.034	<0.001 *
Group	229.251	1	229.251	5.316	0.027 *
Pretest × Group	120.884	1	120.884	2.803	0.102
Deviation	1638.739	38	43.125	-	-

* *p* < 0.05.

**Table 5:** Descriptive statistics of the pretest and posttest results of learning effectiveness.

Group	Number of Subjects		Mean Score		Standard Deviation	
	Pretest	Posttest	Pretest	Posttest	Pretest	Posttest
AR-based learning group	22	22	68.27	91.68	2.944	1.545
Traditional learning group	20	20	71.05	85.75	2.924	2.487

**Table 6:** Summary of ANCOVA analysis results.

Source of Analysis	Type III Sum of Squares	Degree of Freedom	Mean Sum of Squares	*F*-Test	*p*-Value
Pretest score	1692.899	1	1692.899	37.521	<0.001 *
Group	548.132	1	548.132	12.149	0.001 *
Deviation	1759.623	39	45.119		

* *p* < 0.05.

**Table 7:** Marginal mean.

Group	Mean Score	Standard Deviation	95% Confidence Interval	
			Lower Limit	Upper Limit
AR-based learning group	92.321	1.436	89.416	95.225
Traditional learning group	85.047	1.506	82.000	88.094

**Table 8:** Level of significance for the four dimensions of learning motivation.

Dimension of Learning Motivation	*F*-Test	*p*-Value	Mean Value	
			AR-Based Learning Group	Traditional Learning Group
Attention	105.497	<0.001 *	4.52	3.55
Relevance	16.523	<0.001 *	4.26	3.59
Confidence	34.641	<0.001 *	4.70	3.96
Satisfaction	24.789	<0.001 *	4.71	4.08

* *p* < 0.001.

**Table 9:** Level of significance for each question in the Attention dimension.

Question	*F*-Test	*p*-Value	Mean Value		Mean Deviation
			AR-Based Learning Group	Traditional Learning Group	
The instruction and guidance for logic programming training could not arouse my interest in learning.	34.090	<0.001 *	4.45	3.45	1
The learning method used for this logic programming training could attract my attention.	40.666	<0.001 *	4.59	3.60	0.99
The learning method used in this logic programming training was novel to me.	37.594	<0.001 *	4.59	3.65	0.94
In comparison with regular classes, the learning method used in this logic programming training enabled me to stay attentive for a longer time.	24.971	<0.001 *	4.45	3.60	0.85
This learning method used in this logic programming training allowed me to be more focused.	40.539	<0.001 *	4.52	3.55	0.97

* *p* < 0.001.

**Table 10:** Level of significance for each question in the Relevance dimension.

Question	*F*-Test	*p*-Value	Mean Value		Mean Deviation
			AR-Based Learning Group	Traditional Learning Group	
The content of logic programming training is helpful for my future programming learning.	7.729	0.008 *	4.14	3.45	0.69
I cannot connect the content of logic programming training to what I have learned before.	8.592	0.006 *	4.27	3.65	0.62
I am aware of what should be learned in this logic programming training.	22.260	<0.001 **	4.64	3.85	0.79
I can apply the thinking logic learned in this logic programming training to solving real-world problems.	8.592	0.006 *	4.27	3.65	0.62
The knowledge acquired from logic programming training is helpful to me.	9.544	0.004 *	4.00	3.35	0.65

* *p* < 0.01, ** *p* < 0.001.

**Table 11:** Level of significance for each question in the Confidence dimension.

Question	*F*-Test	*p*-Value	Mean Value		Mean Deviation
			AR-Based Learning Group	Traditional Learning Group	
I find that this logic programming training is at an appropriate level of difficulty.	12.839	<0.001 **	4.82	4.20	0.62
I know how to complete the learning tasks in logic programming training.	32.540	<0.001 **	4.64	3.70	0.94
I find the learning model of the logic programming training hard to understand.	15.259	<0.001 **	4.64	3.90	0.74
I have confidence in comprehending all knowledge taught in logic programming training.	8.293	0.006 *	4.55	4.05	0.5
I had excellent learning performance in the logic programming training. I believe this result was achieved through my hard work.	19.721	<0.001 **	4.86	4.10	0.76

* *p* < 0.01, ** *p* < 0.001.

**Table 12:** Level of significance for each question in the Satisfaction dimension.

Question	*F*-Test	*p*-Value	Mean Value		Mean Deviation
			AR-Based Learning Group	Traditional Learning Group	
I am dissatisfied with what I have learned from this logic programming training.	12.928	<0.001 **	4.59	4.00	0.59
I enjoyed a sense of accomplishment when I successfully completed all the tasks in the logic programming training.	7.735	0.008 *	4.68	4.15	0.53
I am very delighted to have experienced this logic programming training activity.	12.337	0.001 *	4.86	4.30	0.56
When experiencing the logic programming training, I felt like time was flying.	10.639	0.002 *	4.68	4.05	0.63
The teaching method used in this logic programming training was novel and fun.	23.359	<0.001 **	4.77	3.90	0.87

* *p* < 0.01, ** *p* < 0.001.

## Data Availability

The data presented in this study are available in this article.
